# Report of One Hundred and Fifty Cases Receiving the Pasteur Treatment at the Georgia Pasteur Institute

**Published:** 1903-11

**Authors:** James N. Brawner

**Affiliations:** Atlanta, Ga., Physician in charge of the Georgia Pasteur Institute, Neurologist to Tabernacle Infirmary and the Hospital for Incurables


### REPORT OF ONE HUNDRED AND FIFTY CASES RECEIVING THE PASTEUR TREATMENT AT THE GEORGIA PASTEUR INSTITUTE.

BY JAMES N. BRAWNER, M.D., ATLANTA, GA.,

Physician in charge of the Georgia Pasteur Institute, Neurologist to Tabernacle Infirmary and the Hospital for Incurables.

Of the one hundred and fifty patients that have received the Pasteur treatment at the Georgia Pasteur Institute twenty-one were bitten on the head, face, and bare neck, the wound being single in seven cases, multiple in ten, and severe and multiple in four. Ninety-six were bitten on the hands, bare legs and feet, the wound being single in forty-two, multiple in forty-one, and severe and multiple in thirteen. Twenty-five were bitten on the body or limbs through the clothing and six were bitten on the body or limbs through the torn clothing. Of those bitten on the face the animal was demonstrated rabid by the inoculation test in eight cases and in three instances by other animals taking the disease from the bite of the same animal. Of those bitten on the hands and bare feet the dog was demonstrated rabid in thirty-nine instances. Of the total number of cases treated the dog was demonstrated rabid by the inoculation of rabbits in fifty-three cases, and by other animals taking the disease in eleven. The dogs had typical symptoms of rabies in eighty-one and escaped observation in six. Three patients have been treated where the dog was demonstrated not rabid by the inoculation test. It will be noticed that of the 150 cases treated, 117 were either bitten on the head, face, hands or bare feet, and not through the clothing.

Forty-five of the patients were men, fourteen women, sixty-four boys and twenty-six girls. As is seen more men and boys are bitten than women or girls.

The youngest patient treated was two years of age, the oldest seventy-eight.

They came from the following States: Georgia, 76; Louisiana, 38; Alabama, 15; South Carolina, 9; Tennessee, 2; North Carolina, 3; Mississippi, 4; and Texas, 1. Ninety-five came from rural districts and fifty-five from cities.

Two of the ladies treated were six and seven months pregnant, respectively, and no bad results followed.

All cases have been treated successfully with the exception of one. This was an old man who was bitten severely in the eye, through the nose and on the lip, and in a region supplied by many nerves. He developed the disease several days after returning home and died with typical symptoms of rabies. One patient, a small negro boy, developed the disease on the tenth day after commencing treatment and died five days after the symptoms first developed. This was before immunity could possibly be established, and, therefore, can not be regarded as a failure. Two patients came to the institute after the symptoms were fully developed and each case died on the day following their arrival.

As to the efficacy of the Pasteur treatment in preventing the development of hydrophobia, much discussion has occurred and opinions are still somewhat divided. So long as we find men in the medical profession who even deny the existence of such a disease it is reasonable to suppose that they will deny the value of the treatment. At the Pasteur Institute in Paris the mortality has averaged about one half of one per cent., and this is about the per cent. lost by all the institutes of which I have a record. Of those not receiving the Pasteur treatment at least 18 per cent. die.

Since the institute has been in operation I have kept a record of all those cases bitten in the Southern States so far as I could by watching the newspapers. During the last three years I know of about 250 people who have been bitten by rabid dogs and those supposed to have been rabid. Of these about 170 have received the Pasteur treatment here and at other institutes, with the record of one death, which I have reported. Of the remaining eighty, which did not receive the treatment but invariably resorted to that magical substance, the madstone, sixteen deaths have occurred to my certain knowledge, or about 20 per cent., which corresponds very nearly with the statistics of other writers. As is shown above, the death rate occurring in those treated is about three fifths of one per cent.

During the last year we have treated thirty-six cases from Georgia and none of these have developed hydrophobia. During the same period about twenty were bitten that did not take the treatment, and among this number four deaths have already occurred.

While the above statistics are small in comparison with some of the institutes, especially of the one at Paris, they lend some weight to the indubitable evidence in favor of the Pasteur treatment for the prevention of rabies.

